# Bark extract of *Cassia sieberiana* DC. (Caesalpiniaceae) displayed good antibacterial activity against MDR gram-negative phenotypes in the presence of phenylalanine-arginine *β*-naphthylamide

**DOI:** 10.1186/s12906-020-03148-3

**Published:** 2020-11-12

**Authors:** Marilene M. M. Ambadiang, Brice C. K. Atontsa, Simplice B. Tankeo, Paul Nayim, Brice E. N. Wamba, Gabin T. M. Bitchagno, James D. S. Mpetga, Veronique B. Penlap, Victor Kuete

**Affiliations:** 1 Department of Biochemistry, University of Dschang, P.O. Box 67, Dschang, Cameroon; 2 Department of Biochemistry, University of Yaounde 1, P.O. Box 812, Cameroun, Yaounde, Cameroon; 3 Department of Chemistry, University of Dschang, P.O. Box 67, Dschang, Cameroon; 4 Institute of Organic Chemistry, University of Mainz, Duesbergweg 10-14, D-55128 Mainz, Germany

**Keywords:** *Cassia sieberiana*, Gram negative bacteria, Multidrug resistance, Efflux pumps, Infectious diseases

## Abstract

**Background:**

Multidrug-resistant (MDR) bacteria remain a major cause of morbidity and mortality globally. The present study was designed to investigate the in vitro antibacterial activities of crude methanol extract and constituents isolated by Column Chromatography (CC) from *Cassia sieberiana* bark (CSB) against ten MDR Gram-negative bacteria, as well as the mechanisms of action of the most active sample.

**Methods:**

The antibacterial activity of the tested samples (extract, the fractions and their compounds isolated by CC and the structures obtained by exploiting ^1^H and ^13^C *Nuclear magnetic resonance* (NMR) spectra) in the presence and absence of an efflux pumps inhibitor, phenylalanine-arginine β-naphthylamide (PAβN), was evaluated using the micro-dilution method. The effects of the most active sample were evaluated on the cell growth kinetic and on the bacterial H^+^-ATPase proton pumps.

**Results:**

Phytochemical composition of the crude extract showed a rather selective distribution of secondary metabolites (presence of polyphenols, tannins, steroids, triterpenes, flavonoids, alkaloids, saponins and absence of anthocyanins, anthraquinones). The tested samples displayed different antibacterial activities with minimal inhibitory concentrations (MICs) ranging from 64 to 512 μg/mL. Crude extract (CS) and fraction CSc showed the highest inhibitory spectra, both inhibiting all of the studied bacteria except *Enterobacter aerogenes* EA27 strain. Fraction CSc exerted bactericidal effects on most bacteria meanwhile, crude extract (CS) and sub-fraction CSc2 exerted bacteriostatic effects. Compounds 1 (spectaline) and 2 (iso-6-cassine) inhibited the growth of 70% (*Escherichia coli* ATCC8739 and AG102, *Klebsiella pneumoniae* ATCC11296, *Enterobacter aerogenes* ATCC13048 and EA27, *Providencia stuartii* ATCC29916, *Pseudomonas aeruginosa* PA01) and 60% (*Escherichia coli* ATCC8739, *Klebsiella pneumoniae* ATCC11296 and KP55, *Providencia stuartii* ATCC29916, *Pseudomonas aeruginosa* PA01 and PA124) of bacteria respectively with MICs ranging from 128 to 512 μg/mL. In the presence of PAβN, the activities of crude extract CS, fraction CAc and sub-fraction CSc2 strongly increased on most bacteria strains as their MICs significantly decreased. Sub-fraction CSc2 inhibited the H^+^-ATPase proton pumps and altered growth kinetic of *Escherichia coli* ATCC8739.

**Conclusion:**

The overall results justify the traditional use of *C. sieberiana* for the treatment of bacterial infections.

**Supplementary Information:**

The online version contains supplementary material available at 10.1186/s12906-020-03148-3.

## Background

Infectious diseases cause 15 million deaths each year, accounting for approximately 27.12% of deaths worldwide [1]. They are involved in about responsible for 560,000 of the 2.7 million neonatal death registered each year [2]. The discovery of antibiotics reduced considerately the mortality rate associated with bacterial infections. Unfortunately, their inappropriate use led to the development of bacterial resistance known as antibio-resistance. Multidrug-resistant (MDR) bacteria remain a major cause of treatment failure leading to more and more morbidity and mortality associated with infectious diseases [3]. Gram-negative bacteria such as many *Enterobacteria* and *Pseudomonas* are the most resistant groups of bacteria [4]. They express their resistance mostly using efflux pumps and the major family implicated in this resistance is the *Resistance Nodulation-Cell Division* (RND) which is a tripartite complex. This situation is alarming because MDR bacteria reduce the amount of antibiotics available for antibiotherapy and are responsible for therapeutic failures, hence leading to an increase in disease burden [3]. The search for new molecules to tackle bacterial antibio-resistance has become imperative. Plant kingdom has a great number of bioactive substances, and good numbers of medicinal plants from the flora of Cameroon have proven their ability to inhibit the growth of Gram-negative bacteria, including MDR phenotypes [5–9]. *C. sieberiana* (Caesalpiniaceae) is widespread in West Africa and is very common in all savannah woodlands or shrubs of the Sudanian zones up to the edge of the Guinean forest in Casamance. This plant is widely used in traditional medicine as an analgesic in dysmenorrhea, body pain in humans, microbial infections and in veterinary medicine [10]. In the present work, we investigated the antibacterial activity of the crude extract from *C. sieberiana* DC (Caesalpiniaceae) and its constituents isolated by CC and the structures obtained by exploiting ^1^H and ^13^C NMR spectra. The role of bacterial efflux pumps in resistance to the tested samples as well as the antibacterial mode of action were also evaluated.

## Methods

### Chemicals

The antibiotic, chloramphenicol (CHL) ≥ 98% was used as reference drug while *p*-iodonitrotetrazolium chloride (INT) and phenylalanine-arginine β-naphthylamide (PAβN) ≥ 97% were used as microbial growth indicator and efflux pumps inhibitor (EPI) respectively. All these chemicals were provided from Sigma-Aldrich, St. Quentin Fallavier, France. Dimethyl sulfoxide (DMSO, Sigma-Aldrich) at the final concentration of 2.5% was used to dissolve the tested samples.

### Plant material and extraction

The bark of *C. sieberiana* DC (Caesalpiniaceae) used in this study was collected in Bandjoun Division, West Region of Cameroon. This plant was identified at the National Herbarium (Yaounde, Cameroon) where a voucher speciment was deposited under the reference number 40152/SFR/CAM/NHC.

The air dried and powdered bark (2.5 kg) was extracted with 5.5 L of methanol (MeOH) 95% for 72 h at room temperature. After filtration using Whatman N°1 filter paper, the filtrate was concentrated in vacuum, under reduced pressure to yield a crude extract (CSB; 195.7 g). It was then conserved at 4 °C for further use.

### Preliminary phytochemical screening

Preliminary phytochemical assay was performed to detect the presence of the major classes of secondary metabolites in the crude extract, namely alkaloids, flavonoids, phenols, saponins, tannins, anthocyanins, anthraquinones, sterols and triterpenes, using a common phytochemical method previously described [11, 12].

### Isolation of the plant constituents

**Fig. 1:**
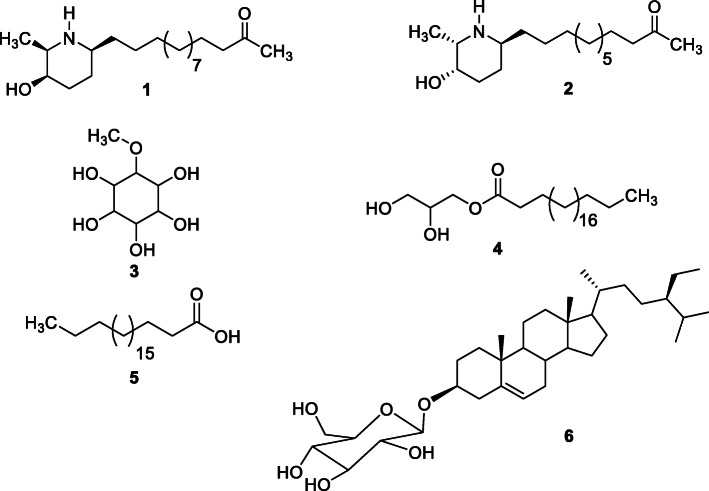
Chemical structures of compounds isolated from *Cassia sieberiana* Spectaline (1), *iso*-6-cassine (2), 3-*O*-methyl-*chiro*-inositol (3), monobehenin (4), arachidic acid (5), sitosterol3-*O*-*β*-D-glucopyranoside (6).

A portion of 167.5 g of CSB was subjected to liquid-liquid extraction by adding ethyl acetate (EtOAc) to the aqueous solution of the crude extract to yield 73.5 g and a residue (90.5 g). The EtOAc extract (70 g) was subjected to silica gel column chromatography (0.200–0.500 mm) eluted with gradients of n-hexane-EtOAc and EtOAc-MeOH as mobile phases (100:0, 90:10, 80:20, 70:30, 60:40 and 50:50). Seventy-five (75) fractions of 300 mL each were collected and combined based on their TLC profiles into three main fractions namely CSBa (20.5 g), CSBb (25.5 g) and CSBc (22.5 g) (see Table S1; supplementary files SF1). Fraction CSBb was purified by column chromatography over silica gel (0.063–0.200 mm) using a gradient of *n*-hexane-EtOAc (100:00, 95:05, 90:10, 85:15, 80:20, 75:25 and 00: 100) to afford arachidic acid (5, 18.0 mg) at *n*-hexane-EtOAc 75:25 and monobehenin (4, 18.0 mg) at *n*-hexane-EtOAc 70:30. Fraction CSBc was purified through silica gel (0.063–0.2 mm) and Sephadex gel (LH-20) chromatography successively and afforded three sub-fractions CSBc1 [(13.5 mg): 7–15], CSBc2 [(11.3 mg): 16–25] and CSBc3 [(12.4 mg): 61–66]. Purification of CSBc was made by column chromatography over silica gel (0.063–0.200 mm) using a gradient of EtOAc-MeOH (100:00, 95:05, 90:10, 85:15, 80:20, 75:25 and 00:100) to afford sitosterol 3-*O*-*β*-D-glucopyranoside (6, 18.0 mg), spectaline (1, 22.0 mg) and *iso*-6-cassine (2, 18.0 mg). The liquid-liquid extraction residue (87.0 g) was subjected to silica gel column chromatography (0.200–0.500 mm) eluted with gradients of EtOAc-MeOH (100:0, 90:10, 80:20, 70:30, 60:40 and 50:50) to afford 3-*O*-methyl-*chiro*-inositol (3, 30.0 mg). Figure 1 shows the chemical structures of the isolated compounds. The ^1^H and ^13^C NMR spectra and major chemical shifts of these compounds are shown in the supplementary files (SF2).

### Antibacterial assay

#### Culture media and bacterial strains

The Mueller Hinton Agar (MHA) was used for the activation of the bacterial strains while Mueller Hinton Broth (MHB) was used for antimicrobial assays.

Ten bacterial strains made of drug sensitive and MDR Gram-negative strains expressing efflux pumps, including reference strains (provided by American Type Culture Collection) and clinical isolates (Laboratory collection) of *Escherichia coli, Pseudomonas aeruginosa, Klebsiella pneumoniae, Enterobacter aerogenes* and *Providencia stuartii*, were used. Their bacterial features CSB are summarized in Table 1. They were maintained on agar slant at 4 °C and cultured on a fresh appropriate agar plate 24 h prior to any antimicrobial test.

**Table 1:** Characteristics of the studied bacterial strains

Species	Types	Characteristics	References
*Escherichia coli*	ATCC 8739	Reference strain	[13]
	AG102	△acrAB mutant AG100, owing acrF gene markedly overexpressed;TET^R^	[13]
*Klebsiella pneumoniae*	ATCC 11296	Reference strain	[14]
	KP55	Clinical MDR isolate Tet^r^, Amp^r^, Atm^r^, Cef^r^	[14]
*Pseudomonas aeruginosa*	PA01	Reference strain	[15]
	PA124	MDR clinical isolate expressing Mex efflux pump	[15]
*Enterobacter aerogenes*	ATCC13048	Reference strain	[13]
	EA 27	Clinical MDR isolate exhibiting energy-dependent norfloxacin and chloramphenicol efflux with Kan^r^, Amp^r^, Nal^r^, Str^r^, Tet^r^	[16]
*Providencia stuartii*	ATCC29916	Reference strain	[17]
	PS 2636	Clinical MDR isolate, AcrAB-TolC	[17]

Ofxa^r^, Kan^r^, Tet^r^, Erm^r^, Amp^r^, Nal^r^,Str^r^, Atm^r^, Cef^r^, Cip^r^, Im/CSB^r^, Chl^r^, Gen^r^, Nis^r^, Flx^r^: resistant (r) to ofloxacin, kanamycin, tetracyclin, erythromycin, ampicillin, nalidixic acid, streptomycin, aztreoname, cefepim, ciprofloxacin, imipenem/Cilastatin sodium, chloramphenicol, gentamycin, nisine and flomoxef respectively; MDR: Multidrug-resistant;*. AcrAB-TolC, AcrAB and Mar A* are efflux pumps

#### Bacterial susceptibility determination

The minimal inhibitory concentrations (MIC) of samples on the studied bacteria strains were determined using a rapid INT colorimetric assay [18, 19]. Briefly, the test samples were first dissolved in DMSO/MHB mixture. The obtained solution was then added to MHB, and serial twofold dilutions carried out (in a 96-well microplate). One hundred microliters (100 μL) of bacterial inoculum (1.5 × 10^8^ CFU/mL) were then added. The plates were covered with a sterile plate sealer, then agitated to mix the contents in the wells using a shaker and incubated at 37 °C for 18 h. The final concentration of DMSO was lower than 2.5% and does not affect microbial growth. Wells containing MHB, 100 μL of inoculums, and DMSO at a final concentration of 2.5% served as a negative control. Chloramphenicol was used as reference antibiotic. The MIC of samples were detected after 18 h of incubation at 37 °C, following addition (40 μL) of 0.2 mg/mL INT and incubation at 37 °C for 30 min [20]. Viable bacteria reduced the yellow dye to pink. MIC was defined as the lowest sample concentration that prevented this change and exhibited complete inhibition of microbial growth. To determine the minimal bactericidal concentrations (MBC), a volume of 150 μL of MHB was introduced in a new 96-well microplate, following addition of 50 μL of the previous well’s microplate contents where no microbial growth was observed and which did not receive the INT (during the reading of the MIC). After 48 h incubation at 37 °C, the MBC of each sample was determined and defined by adding 40 μL of 0.2 mg/mL INT as previously described. Samples were tested alone and latter, in the presence of PAβN, an efflux pump inhibitor, at 30 mg/L final concentration. In this last case, the activity improvement factors (AIFs) were determined to qualify the potentiation level of this inhibitor on the activity of sample, using the _MICsample alone_/_MICsample-PAβN combination_ ratio. All assays were performed in triplicate and repeated thrice.

### Antibacterial mechanisms of action

#### Effect of sub-fraction CSBc2 on bacterial growth kinetic

Bacterial growth kinetic study of sub-fraction CSBc2 which showed the best antibacterial activity was done using a spectrophotometer at 600 nm wavelength [21]. Bacterium used for this study was a reference strain *Escherichia coli* ATCC8739 and the sample was tested at the concentrations of MIC/2, MIC and 2MIC. Firstly, 500 μL of bacterial suspension (1.5 × 10^8^ UFC/ml) from preculture were added to 450 mL MHB (1/100 v/v dilution) and incubated at 37 °C for 18 h under magnetic agitation and in the presence of tested sample at different concentrations. Chloramphenicol was used as positive control whereas, inoculum (1.5 × 10^8^ UFC/mL)/DMSO (2.5% v/v) mixture constituted the negative control. At 0, 0.5, 1 and 2 h followed by regular interval time of 2 h from 2 to 18 h, aliquots of 1 mL from the preparation were deducted and introduced in a spectrophotometric tab and then, the optical density (OD) was read at a wavelength of 600 nm. The bacterial growth curves [OD = f (time)] were plotted using Microsoft Excel software (Fig. 1).

#### Effect of sub-fraction CSBc2 on bacterial H^+^-ATPase-dependent proton pumps

**Fig. 2:**
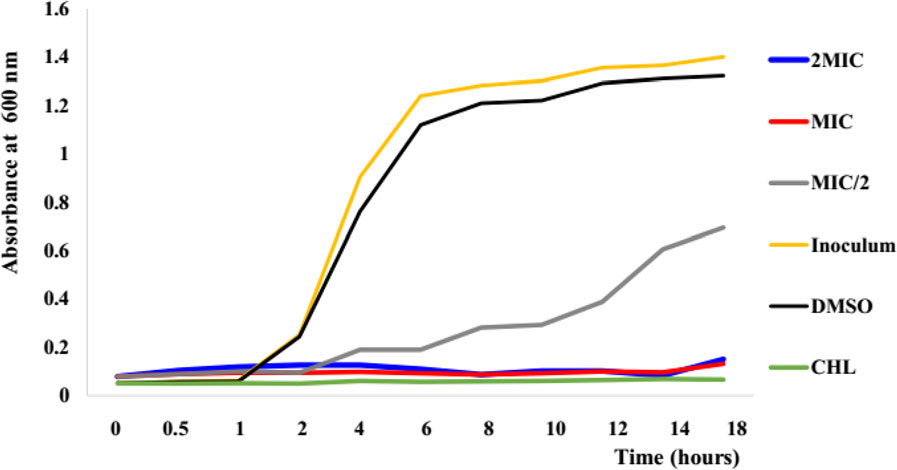
Effect of sub-fraction CSBc2 at different concentrations on growth kinetic of *Escherichia coli* ATCC8739

The ability of sub-fraction CSBc2 to inhibit bacterial H^+^-ATPase-dependent proton pumps was evaluated, by assessing the acidification of the bacterial external environment, through pH measurement, as previously described [22]. Hence, a fresh bacterial colony was dissolved in 20 mL of MHB culture medium and incubated at 37 °C under magnetic agitation for 18 h. Aliquots of 1 mL from this bacterial preculture were deducted and added to MHB to afford 100 mL final volume (1/100 v/v dilution), and then re-incubated at 37 °C for 18 h under magnetic agitation. One hundred milliliter (100 mL) from this bacterial culture was centrifuged at 4000 rds/min for 30 min at 4 °C. Recuperated gut was washed with sterile distilled water then with KCl 50 mM and was dissolved in 50 mL KCl 50 mM. The obtained bacterial suspension was conserved at 4 °C for 18 h (for glucose starvation), after which the pH was adjusted to 7.3 by adding HCl or NaOH solution. Then, 0.5 mL of tested sample was added to 4 mL of this bacterial culture and the mixture was incubated at 37 °C for 10 min, after which, 0.5 mL of glucose 20% was added in order to initiate the acidification of the environment. Inoculum (1.5 × 10^8^ UFC/mL)/DMSO (2.5% v/v) mixture constituted the negative control. The pH values of tested samples at different concentrations were read at room temperature (25 °C) each 10 min for 1 h, using a pH-meter. The curves [pH = f (time)] were labeled using Microsoft Excel software (Fig. 2).

### Data analysis

The plant crude extract, fractions and sub-fractions were considered significantly active when their MIC values were below to 100 μg/mL, moderately active when the MIC values were found between 100 and 625 μg/mL and poorly active when the MIC values were above 625 μg/mL; for antibiotics and isolated compounds, sample with MIC≤10 μg/mL, 10 < MIC≤100 μg/mL or MIC> 100 μg/mL is considered to have strong, moderate or weak activity respectively [23]. When MBC/MIC ratio was ≤4, tested samples were considered as bactericidal and when this ratio was > 4, samples were considered as bacteriostatic [24]. During the evaluation of the antibacterial activity in the presence or absence of an efflux pumps inhibitor, the activity improvement factors (AIFs) were calculated. The activity of an extract, fraction or sub-fraction was considered to be improved when AIF was ≥2 [25].

## Results

### Antibacterial activity of crude extract, fractions and compounds

The antibacterial susceptibility of tested samples was carried determining the MIC and MBC of each sample on studied bacteria (Tables 2, 3 and 4). Bactericidal or bacteriostatic effects of each sample on a bacterial strains were determined by calculating the MBC/MIC ratio. Results presented in Table 2 show that crude extract CSB and fraction CSBc were the best botanicals since they displayed recordable MIC against 90% (9/10) of studied strains with values ranging from 128 to 512 μg/mL. They were not active only against *Enterobacter aerogenes* EA27 and their best activity was against *Klebsiella pneumoniae* KP55. Fractions CSBa and CSBb which were less active inhibited the growth of 30% (3/10) and 20% (2/10) of bacteria respectively. All these samples showed a bactericidal effect against susceptible bacterial strains. In Table 3, only sub-fraction CSBc2 inhibited the growth of many bacteria (70% (7/10)) with MIC ranging from 128 to 256 μg/mL. The other sub-fractions displayed antibacterial activities against 30% (3/10) of studied bacteria. All sub-fractions had bacteriostatic effects against all tested bacteria, excepted sub-fraction CSBc2 which showed bactericidal effect against *Escherichia coli* ATCC8739 and *Pseudomonas aeruginosa* PA124. Furthermore, only compounds 1 and 2 of the six isolated compounds showed antibacterial activity respectively on 70% (7/10) and 60% (6/10) of strains with MIC ranging from 128 to 512 μg/mL (Table 4). Compound 1 exhibited a bactericidal effect against *Escherichia coli* ATCC8739 and *Enterobacter aerogenes* EA27 while compound 2 showed this effect against *Escherichia coli* ATCC8739. Other compounds showed weak or no activity against studied bacteria.

**Table 2:** Minimal inhibitory and bactericidal concentrations of crude extract, his derived fractions and chloramphenicol

Bacterial strains	Tested samples and concentrations (μg/mL)														
	CSB			CSBa			CSBb			CSBc			Chloramphenicol		
	MIC	MBC	R	MIC	MBC	R	MIC	MBC	R	MIC	MBC	R	MIC	MBC	R
*Escherichia coli*															
ATCC8739	512	512	1	512	512	1	–	nt	nd	128	128	1	2	64	32
AG102	512	512	1	–	nt	nd	–	nt	nd	512	512	1	32	256	8
*Klebsiella pneumoniae*															
ATCC11296	512	–	> 1	512	512	1	–	nt	nd	512	–	> 1	32	256	8
KP55	128	512	4	512	512	1	–	nt	nd	128	512	4	64	256	4
*Enterobacter aerogenes*															
ATCC13048	512	512	1	–	nt	nd	–	nt	nd	512	512	1	16	128	8
EA27	–	nt	nd	–	nt	nd	–	nt	nd	–	nt	nd	32	256	8
*Providencia stuartii*															
ATCC29916	512	512	1	–	nt	nd	512	512	1	512	512	1	64	256	4
PS2636	512	512	1	–	nt	nd	512	512	1	512	512	1	64	256	4
*Pseudomonas aeruginosa*															
PA01	512	–	> 1	–	nt	nd	–	nt	nd	512	–	> 1	64	–	> 4
PA124	512	512	1	–	nt	nd	–	nt	nd	512	512	1	64	–	> 4
PSBS (%)	90			30			20			90			100		

*MIC* minimal inhibitory concentration, *MBC* minimal bactericidal concentration R: MBC / MIC ratio (a sample is considered as bacteriostatic or bactericidal when this ratio is > 4 or ≤ 4 respectively) (−): MIC or MBC > 512 μg/mL for crude extract and fractions and > 256 for chloramphenicol nt: not tested nd: not determined (as no MIC and MBC values were not observed till 512 μg/mL) PBSS: percentage of susceptible bacteria to substances CSB is crude extract of *Cassia sieberiana* CSBa, CSBb and CSBc are fractions from CSB

**Table 3:** Minimal inhibitory and bactericidal concentrations of different sub-fractions

Bacterial strains	Sub-fractions and concentrations (μg/mL)								
	CSBc1			CSBc2			CSBc3		
	MIC	MBC	R	MIC	MBC	R	MIC	MBC	R
***Escherichia coli***									
ATCC8739	256	–	> 2	64	64	1	256	–	> 2
AG102	–	nt	nd	256	–	> 2	64	–	> 8
*Klebsiella pneumoniae*									
ATCC11296	64	–	> 8	128	–	> 4	–	nt	nd
KP55	–	nt	nd	–	nt	nd	–	nt	nd
*Enterobacter aerogenes*									
ATCC13048	512	–	> 1	256	–	> 2	–	nt	nd
EA27	–	nt	nd	–	nt	nd	–	nt	nd
*Providencia stuartii*									
ATCC29916	–	nt	nd	256	–	> 2	256	–	> 2
PS2636	–	nt	nd	256	–	> 2	–	nt	nd
*Pseudomonas aeruginosa*									
PA01	–	nt	nd	–	nt	nd	–	nt	nd
PA124	–	nt	nd	128	128	1	–	nt	nd
PSBS (%)	30			70			30		

*MIC* minimal inhibitory concentration, *MBC* minimal bactericidal concentration R: MBC / MIC ratio (a sample is considered as bacteriostatic or bactericidal when this ratio is > 4 or ≤ 4 respectively) (−): MIC or MBC > 512 μg/mL nt: not tested nd: not determined (as no MIC and MBC values were not observed till 512 μg/mL) PBSS: percentage of susceptible bacteria to substances CSBc1, CSBc2 and CSBc3 are sub-fractions from fraction CSBc

**Table 4:** Minimal inhibitory and bactericidal concentrations of different compounds

Bacterial strains	Compounds and concentrations (μg/mL)											
	1			2			6			Chloramphenicol		
	MIC	MBC	R	MIC	MBC	R	MIC	MBC	R	MIC	MBC	R
*Escherichia coli*												
ATCC8739	128	512	4	256	–	> 1	256	–	> 2	2	64	32
AG102	256	–	> 1	–	nt	nd	–	nt	nd	32	256	8
*Klebsiella pneumoniae*												
ATCC11296	128	nt	nd	256	512	2	–	nt	nd	32	256	8
KP55	–	–	> 1	512	–	> 1	–	nt	nd	64	256	4
*Enterobacter aerogenes*												
ATCC13048	128	–	> 1	–	nt	nd	–	nt	nd	16	128	8
EA27	128	512	4	–	nt	nd	–	nt	nd	32	256	8
*Providencia stuartii*												
ATCC29916	128	–	> 1	256	–	> 2	–	nt	nd	64	256	4
PS2636	–	nt	nd	–	nt	nd	–	nt	nd	64	256	4
*Pseudomonas aeruginosa*												
PA01	512	–	> 1	512	–	> 1	512	nt	nd	64	–	> 4
PA124	–	nt	nd	512	–	> 1	–	nt	nd	64	–	> 4
PSBS (%)	70			60			20			100		

*MIC* minimal inhibitory concentration, *MBC* minimal bactericidal concentration R: MBC / MIC ratio (a sample is considered as bacteriostatic or bactericidal when this ratio is > 4 or ≤ 4 respectively) (−): MIC or MBC > 512 μg/mL nt: not tested nd: not determined (as no MIC and MBC values were not observed till 512 μg/mL) PBSS: percentage of susceptible bacteria to substances Compounds **1**, **2** and **6** were obtained from fraction CSBc Compounds **3** isolated from the residual extrac**t** and compounds **4** and **5** isolated from fraction CSBb did not showed any activity against all studied bacterial

Samples were also tested in presence of an efflux pump inhibitor in order to determine their role in the mechanism of resistance of studied bacterial strains (Table 5).

**Table 5:** Minimal inhibitory concentrations of tested samples in presence of PAβN

Bacterial strains	Tested samples and concentrations (μg/mL)																	
	CSB			CSBc			CSBc1			CSBc2			CSBc3			Chloramphenicol		
	MIC	+PAβN	R	MIC	+PAβN	R	MIC	+PAβN	R	MIC	+PAβN	R	MIC	+PAβN	R	MIC	+PAβN	R
*Escherichia coli*																		
ATCC8739	512	64	8	512	16	32	256	16	16	256	64	4	256	256	1	2	< 0,5	> 4
AG102	512	64	8	512	32	16	–	–	nd	256	< 4	> 64	64	–	> 8	32	4	8
*Klebsiella pneumoniae*																		
ATCC11296	512	–	< 1	512	128	4	64	16	4	256	128	2	–	< 4	> 128	32	8	4
KP55	128	< 4	> 32	128	256	0.5	–	–	nd	–	256	> 2	–	–	nd	64	32	2
*Enterobacter aerogenes*																		
ATCC13048	512	16	32	512	256	2	512	–	< 1	256	32	8	–	< 4	> 128	16	32	2
EA27	–	32	> 16	–	< 4	128	–	64	> 8	–	256	> 2	–	–	nd	32	16	2
*Providencia stuartii*																		
ATCC29916	512	32	16	512	256	2	–	–	nd	256	128	2	256	8	32	64	8	8
PS2636	512	32	16	512	128	4	–	–	nd	256	–	< 2	–	–	nd	64	16	4
*Pseudomonas aeruginosa*																		
PA01	512	–	nd	512	–	< 1	–	–	nd	–	–	nd	–	< 4	> 128	64	8	8
PA124	512	–	nd	512	256	2	–	–	nd	128	256	0,5	–	16	> 32	256	16	16
PIA (%)		70			80			30			70			60			100	

*MIC* minimal inhibitory concentration R = AIF: MICample alone / MICample_+PAβN_ ratio (this means the factor which determine the improvement of the activity of samples by PAβN; the activity of a sample was considered to be improved when its AIF was > 2). (+ PAβN): represent MIC values of tested samples obtained in presence of PAβN (−): MIC > 512 μg/mL for crude extract, fractions and sub-fractions PIA: percentage of improved activity nd: not determined (as no MIC values were not observed till 512 μg/mL) CSB: crude extract CSBc: fraction CSBc1, CSBc2 and CSBc3 are sub-fractions from fraction CSBc

### Role of efflux pumps on the resistance of bacteria to the tested samples

In the presence of PAβN (Table 5), the activity of all tested samples against many bacteria increased considerably. The activities of crude extract CSB, fraction CSAc, sub-fraction CSBc2 and sub-fraction CSBc3 were improved respectively on 70% (7/10), 80% (8/10), 70% (7/10) and 60% (6/10) of studied bacteria, AIFs ranged from 2 to > 128. The activity of sub-fraction CSBc1 was improved only against 30% (3/10) of bacteria. *Pseudomonas aeruginosa* strains were more sensitive vis-a-vis chloramphenicol and sub-fraction CSBc3 and more resistant towards other samples, whereas *Escherichia coli* strains were resistant towards these two substances (chloramphenicol and sub-fraction CSBc3) and sensitive towards other samples.

### Second metabolites classes detected in the crude extract

Phytochemical analysis of *C. sieberiana* crude extract showed the presence of polyphenols, flavonoids, tannins, alkaloids, saponins, steroids and triterpenes. Anthraquinones and anthocyanin were absent (Table 6).

**Table 6:** Phytochemical composition of *Cassia sieberiana* bark

Metabolite classes	Crude extract (CSB) composition
Polyphenols	+
Flavonoids	+
Tannins	+
Anthraquinones	–
Alkaloids	+
Saponins	+
Steroids	+
Triterpenes	+
Anthocyanins	–

(+): presence of metabolites (−): absence of metabolites

### Effect of sub-fraction CSBc2 on the bacterial growth kinetic

Data depicted in Fig. 2 show the growth of *Escherichia coli* ATCC8739 in absence and presence of sub-fraction CSBc2 at different concentrations (MIC/2, MIC and 2 x MIC) and chloramphenicol (at MIC) during a time range. This figure showed three stages (lag, exponential and stationary stages) of bacterial growth for negative controls (inoculum and DMSO 2.5%). Lag phase is prolonged till the 18th hour in the presence of sub-fraction CSBc2 at MIC and 2 x MIC, as well as in the presence of a reference antibiotic, chloramphenicol (CHL). At MIC/2 of sub-fraction CSBc2, this phase persisted for 4 h before exponential growth phase started.

### Effect of sub-fraction CSBc2 on bacterial H^+^-ATPase-dependent proton pumps

**Fig. 3:**
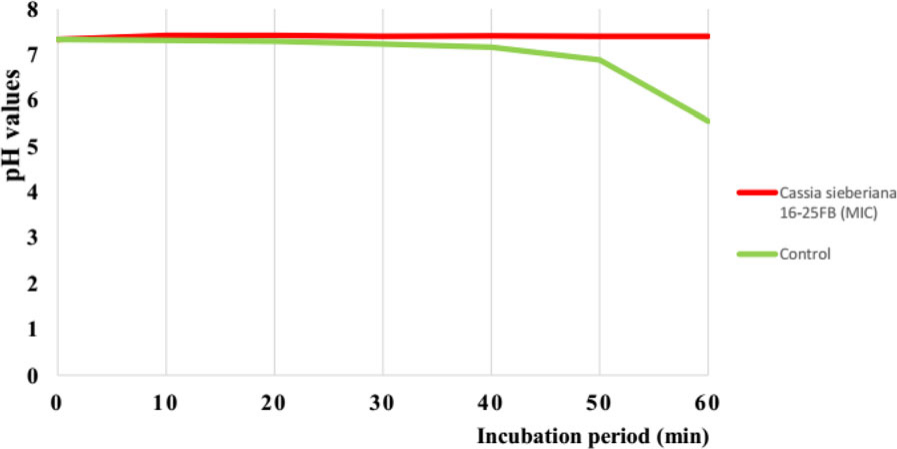
Effect of sub-fraction CSBc2 on *Escherichia coli* ATCC8739 H^+^-ATPase- dependent proton pumps

Figure 3 shows the variation of pH (acidity) of cell culture containing *Escherichia coli* ATCC8739, at different incubation periods in presence of sub-fraction CSBc2 at MIC concentration. DMSO 2.5% was used as negative control. The curves obtained showed that after 60 min of the experiment, the pH values in the presence of sub-fraction CSBc2 remained almost constant from the beginning to the end of the experiment (7.33). Meanwhile in presence of control (DMSO 2.5%), pH values considerably decreased (from 7.33 to 5.5).

## Discussion

The phytochemical screening of CSB showed the presence of some classes of secondary metabolites including polyphenols, alkaloids, flavonoids, saponins, tannins, steroids and triterpenes. The obtained results are in accordance with previous studies done by other authors on *C. sieberiana* bark [26–33]. The secondary metabolites contained in several medicinal dietary plants are known for their pharmacological activities including antibacterial properties [34, 35].

Following established cutoff points [23], a botanical is considered significantly active when MIC< 100 μg/mL, moderately active when 100 ≤ MIC≤625 μg/mL and poorly active when MIC> 625 μg/mL. Based on these cutoff points, some of the tested samples (CSBc1, CSBc2, CSBc3) can be considered significantly active, with MIC value of 64 μg/mL, while CSB generally displayed a moderate activity on 90% of studied bacterial strains. The phytochemical composition of CS can be responsible for the antibacterial activity of the samples, since tannins, saponins, flavonoids and steroids (reported in this fraction) are known to have antimicrobial and curative properties against several pathogens [31]. The results of antimicrobial activity obtained with the crude extract of CSB in this study corroborate those of [36] who reported the activity of the methanol extract of this plant against sensitive Gram-negative and Gram-positive bacteria, and attributed this activity to the presence of flavonoids. The MICs variability from one sample to another, could be explained either by the difference in their secondary metabolite’s contents, the quantity or the quality of these metabolites [7, 37, 38].

Multidrug-resistant Gram-negative pathogens are global public health concern as therapeutic options for treating such infections are dwindling. In view of the scarcity of new antibiotics, a parallel strategy to the discovery of new molecules is the use of substances inhibiting bacterial resistant pathways. Therefore, only three compounds (the two alkaloids spectaline (1) and iso-6-cassine (2) and sitosterol 3*-O-β-*D-glucopyranoside (6)) isolated from fraction CSBc which was the most active fraction, inhibited the growth of some of the studied bacteria, but with weak activities. Indeed, (−)-*iso*-6-spectaline has been reported to exhibit a weak antimicrobial activity against *Staphylococcus epidermidis* [39]. Cassine isolated from *Senna racemosa* Mill. leaves (Fabaceae) showed weak antimicrobial activities against *Staphylococcus aureus*, *Bacillus subtilis* and *Candida albicans* [40]. Furthermore, a mixture compounds of (−)-spectaline and (−)-cassein from the flower of *Senna spectabilis* (DC.) Irwin & Barneby (Fabaceae) has been reported to have anti-leishmanial properties [41]. However, to the best of our knowledge, no antibacterial study against MDR Gram-negative strains has been reported so far with these compounds.

It can be noticed from data reported in Table 5 that, efflux pumps could be responsible for the low action of some tested samples such as CSB, fraction CSBc and sub-fraction CSBc2. In effect, the modulation of their activities on some studied bacterial strains demonstrates that bioactive compounds responsible for their antibacterial activity are efflux pumps substrates and might therefore have an intracellular action site, and thus, PAβN has effectively prevented the proper functioning of these efflux pumps. It can also be noted that fraction CSBc had the best modulating effect since its MICs were shown to decrease on almost all the studied bacterial strains. These results corroborate some previously reported data [13, 42] stating that, efflux mechanism can be blocked by various efflux pump inhibitors and this will restore the intracellular concentrations as well as the activities of antimicrobial substances. Phenylalanine arginine *β*-naphthylamide, used as an efflux pump inhibitor, is one of the best efflux pump inhibitors and it particularly acts on RND pumps [43–45]. The activities of some tested samples were not improved in the presence of PAβN. This could result from the fact that they were not efflux pump substrates or that the efflux mechanism was not the only mechanism expressed in studied bacteria. Therefore, *Pseudomonas aeroginosa* strains were more resistant to most of the tested sample-PAβN combinations. It was shown that *P. aeroginosa* is a highly pathogenic microorganism responsible for multiple infections in both humans and plants. Efflux-mediated antibiotic resistance in *P. aeruginosa* is conferred primarily by efflux pumps belonging to the resistance nodulation-cell division (RND) superfamily that extrudes a broad spectrum of antimicrobial compounds and other substrates [46, 47].

Analysis of curves in Fig. 2 showed that sub-fraction CSBc2 exerted its inhibitory activity at lag phase of bacterial growth depending on the concentrations. The progress of the latent phase was disturbed when the microorganism encountered sub-fraction CSBc2, since an extension of this phase at MIC and 2 x MIC was observed throughout the experiment period. These results corroborate those obtained earlier [48], showing that the lag phase is associated with biosynthesis of many enzymes which helps a microorganism to adapt with its new environment with respect to nutritive elements present, and that any distortion of this process causes the lag phase to be prolonged. Net growth of the bacterial strains treated the latter with tested sample at MIC/2 was observed due to the presence of the molecules responsible for the antibacterial activity at a sub-inhibitory concentration. The relatively low decrease in the number of bacterial colonies after the experiment gives information on the bacteriostatic effect of the extract. This can be confirmed with the higher MBC values observed.

Sub-fraction CSBc2 was also tested on H + -ATPase-dependent proton pumps of *E. coli* ATCC8739 as well as on its potential to inhibit the cell growth (Fig. 3). Indeed, the energy required for the development of the metabolic reactions of the bacteria depends on the proper functioning of these H + -ATPase-dependent proton pumps which are protein enzymes necessary for the formation of a large electrochemical gradient of protons and the maintenance of the intracellular pH [49]. The inhibition of the proton pumps will thus become lethal to the bacterium. The inhibition of these pumps by a substance therefore leads to a decrease in H^+^ protons in the medium which becomes less and less acidic, indicating the inactivation of the H + -ATPase pumps, compromising the bacterium survival that will die for lack of energy [49]. This confirmed the killing effect of sub-fraction CSBc2 on *E. coli* ATCC8739.

The data obtained in this experiment therefore indicates that sub-fraction CSBc2 is a potential proton pump inhibitor and corroborates those obtained by [50] revealing that proton-ATPase pumps are responsible for regulating the cytoplasm and that any inhibition of this pump equally inhibits bacterial growth. It is well known that bacteria are viable in a wide range of pH (1–11) and that the bacterial cytoplasmic pH is kept near neutral. It is also generally accepted that bacterial cytoplasmic pH is regulated by various cation transport systems [49]. Some data suggest that in *E. coli*, cytoplasmic pH is regulated by proton extrusion via the respiratory chain and potassium influx at acid pH, and cation/proton antiporter regulates the pH in alkaline states [50].

## Conclusion

This work was aimed at contributing to the fight against infectious diseases caused by MDR bacteria phenotypes. Crude extract CSB, fraction CSBc and sub-fraction CSBc2 exhibited moderate antibacterial activities on several strains and their activities significantly increased in the presence of PAβN. Sub-fraction CSBc2 inhibited the cell growth at sub-inhibitory concentrations as well as bacterial H^+^-ATPases-dependent proton pumps. Compounds 1 (spectaline) was most active than other compounds. Results obtained validate the therapeutic usage of *C. sieberiana* bark in traditional medicine and indicate that their bioactive constituents can be combined to an efflux pumps inhibitor to overcome the problem of bacterial resistance involving efflux pumps. The mechanisms of action of this plant are reported for the first time in the present work. However, further studies about the combination of these plant constituents with commonly used antibiotics are important to efficiently fight against infectious diseases.

## Supplementary Information

**Additional file 1:** **Supplementary file.** SF 1. Tables showing the fractionation and purification of *C. sieberiana* bark; SF 2. ^1^H and ^13^C NMR and major chemical shifts of studied compounds; G1. Data and graphic for the effect of sub-fraction CSBc2 on the bacterial growth kinetic; G2. Data and graphic for the effect of sub-fraction CSBc2 on bacterial H^+^-ATPase-depending proton pumps.

## Data Availability

All data generated or analyzed during this study are included in this published article and its supplementary information files.

## Abbreviations

CSB: *Cassia sieberiana*
ATCC: American Type Culture Collection
MIC: Minimal inhibitory concentration
MBC: Minimal bactericidal concentration
DMSO: Dimethylsulfoxide
INT: p-Iodonitrotetrazolium chloride
PAβN: Phenylalanine arginine-β- naphthylamide
SFR/CAM: Society of forest reserve of Cameroon
NHC: National herbarium of Cameroon
CHL: Chloramphenicol
MHA: Mueller Hinton agar
MHB: Mueller Hinton broth
OD: Optical density
RND: Resistance-nodulation-cell division
EPI: Efflux pumps inhibitor
AIF: Activity improvement factor
*NMR*: *Nuclear magnetic resonance*
CC: Column Chromatography
