# Supplementary material for: Bark extract of Cassia sieberiana DC. (Caesalpiniaceae) displayed good antibacterial activity against MDR gram-negative phenotypes in the presence of phenylalanine-arginine β-naphthylamide
Source: BMC Complement Med Ther. 2020 Nov 12;20:342. doi: 10.1186/s12906-020-03148-3 (PMC7664092; doi:10.1186/s12906-020-03148-3)
Supplement: Supplementary file 1 — Additional file 1: Supplementary file. SF 1. Tables showing the fractionation and purification of C. sieberiana bark; SF 2. 1H and 13C NMR and major chemical shifts of studied compounds; G1. Data and graphic for the effect of sub-fraction CSBc2 on the bacterial growth kinetic; G2. Data and graphic for the effect of sub-fraction CSBc2 on bacterial H+-ATPase-depending proton pumps. [file 12906_2020_3148_MOESM1_ESM.doc]

**Botanicals from the bark of *Cassia sieberiana* (Caesalpiniaceae) displayed good antibacterial activity against MDR Gram-negative phenotypes in the presence of an efflux pumps inhibitor, phenylalanine-arginine β-naphthylamide**

Marilene M. M. Ambadiang^1,2^, Brice C. K. Atontsa^3^, Simplice B. Tankeo^1^, Paul Nayim^1^, Brice E.N. Wamba^1^, Gabin T. M. Bitchagno^3,4^, James D.S. Mpetga^3^, Veronique B. Penlap^2^ and Victor Kuete^1*^

*^1^Department of Biochemistry, University of Dschang, P.O. Box 67, Dschang, Cameroon*

*^2^Department of Biochemistry, University of Yaounde 1, Cameroun P.O. Box 812, Yaounde, Cameroon*

*^3^Department of Chemistry, University of Dschang, P.O. Box 67, Dschang, Cameroon*

*^4^Institute of Organic Chemistry, University of Mainz, Duesbergweg 10-14, D-55128 Mainz, Germany*

*Corresponding author:

*Phone: +237 677355728; email:* [*kuetevictor@yahoo.fr*](mailto:kuetevictor@yahoo.fr) *(Prof. Dr. Victor Kuete)*

Author’s e-mails:

*Marilene M. M. Ambadiang:* [*mae.marie1995@yahoo.com*](mailto:mae.marie1995@yahoo.com)

*Brice C. K. Atontsa:* [*briceatontsa@gmail.com*](mailto:briceatontsa@gmail.com)

*Simplice B. Tankeo:* [*simplicetankeo@yahoo.fr*](mailto:simplicetankeo@yahoo.fr)

*Paul Nayim :* [*paulnayim@gmail.com*](mailto:paulnayim@gmail.com)

*Brice E.N. Wamba:* [*wambaelvis@yahoo.fr*](mailto:wambaelvis@yahoo.fr)

*Gabin T. M. Bitchagno:* [*bmgt198716@ymail.com*](mailto:bmgt198716@ymail.com)

*James D.S. Mpetga :* [*james.mpetga@univ-dschang.org*](mailto:james.mpetga@univ-dschang.org)

*Veronique B. Penlap :* [*vpenlap@yahoo.fr*](mailto:vpenlap@yahoo.fr)

*Victor Kuete :* [*kuetevictor@yahoo.fr*](mailto:kuetevictor@yahoo.fr)

**Supplementary files**

**SF 1.** Tables showing the fractionation and purification of *Cassia sieberiana* bark

**Table S1.** Fractionation of crude extract (CS)

| **Eluent** | **Sub-fractions** | **Fractions grouping** |
| --- | --- | --- |
| n-hexane-EtOAc |  |  |
| 100 : 00 | 1 - 7 | 1 - 13 (CSa) |
| 90 : 10 | 8 - 15 |
| 80 : 20 | 16 - 25 | 14 - 50 (CSb) |
| 70 : 30 | 26 - 35 | 51 - 75 (CSc) |
| 60 : 40 | 36 - 45 |
| 50 : 50 | 46 - 54 |
| 00 : 100 | 55 - 65 |
| MeOH |  |
| 100 : 00 | 66 - 75 |

**Table S2.** Purification of fraction CSc

| **Eluent** | **Remarks and isolated compounds** |
| --- | --- |
| n-hexane-EtOAc |  |
| 100 : 00 | Complex mixture |
| 95 : 05 |
| 85 : 15 |  |
| 80 : 20 |  |
| 75 : 25 | Arachidic acid (**5**, 18.0 mg) |
| 70 : 30 | monohederin (**4**, 18.0 mg) |
| MeOH |  |
| 100 : 00 | Complex mixture |

**Table S3.** Purification of fraction CSc

| **Eluent** | **Remarks and isolated compounds** |
| --- | --- |
| EtOAc-MeOH |  |
| 100 : 00 | sitosterol 3-*O*-*β*-D-glucopyranoside (**6**, 18.0 mg) |
| 95 : 05 |
| 85 : 15 | spectaline (**1**, 22.0 mg) and *iso*-6-cassine (**2**, 18.0 mg) |
| 80 : 20 | Complex mixture |
| 75 : 25 |
| 70 : 30 |
| MeOH |  |
| 100 : 00 | Complex mixture |

**Table S4**. Purification of the liquid-liquid extraction residue

| **Eluent** | **Remarks and isolated compounds** |
| --- | --- |
| EtOAc-MeOH |  |
| 100 : 00 | Complex mixture |
| 90 : 10 |
| 80 : 20 |
| 70 : 30 | 3-*O*-methyl-*chiro*-inositol (**3**, 30.0 mg). |
| 60 : 40 | Complex mixture |
| 50 : 50 |
| MeOH |
| 100 : 00 |

**SF 2.** ^1^H and ^13^C NMR and major chemical shifts of studied compounds

- Compound **1** (spectaline)

White amorphous solid ^13^C NMR (150 MHz, DMSO-*d_6_*+CDCl_3_): *δ* (ppm) 208.7 (C-13’), 64.8 (C-3), 57.3 (C-6), 56.1 (C-2), 43.6 (C-12’), 33.2 (C-1’), 30.3 (C-4), 30.0 (C-14’); 29.0 - 29.5 (C-2’-9’), 25.3 (C-10’); 23.7 (C-11’); 22.2 (C-5), 15.8 (C-1’’). ^1^H NMR (600 MHz, DMSO-*d_6_*+CDCl_3_): *δ* (ppm) 8.02 (1H, s, H-1), 5.21 (1H, brs, OH-3), 3.74 (1H, s, H-3), 3.04 (1H, s, H-2), 2.82 (2H, s, H-6), 2.35 (2H, t, *J* = 6.0 Hz, H-12’), 2.04 (3H, s, H-14’), 1.90 (1H, dd, *J* = 18.0 and 6.0 Hz, H-4*α*), 1.70 (1H, m, H-1’*α*), 1.65 (2H, m, H-5), 1.57 (1H, dd, *J* =18.0 and 6.0 Hz, H-4*β*), 1.47 (1H, m, H-1’*β*), 1.43 (2H, m, H-11’), 1.27 (3H, d, *J* = 12.0 Hz, H-1”), 1.16 - 1.23 (18H, brs, H-2’-10’).


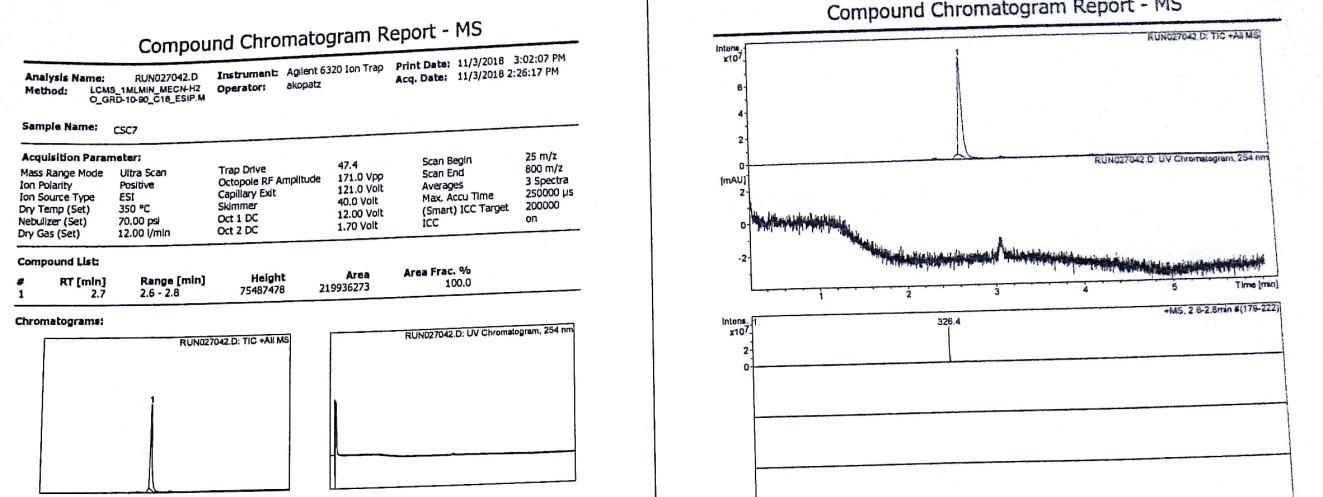


Figure S1: Full LC-MS profile of **1**

Figure S2: Full ^13^C NMR spectrum (DMSO-*d_6_*+CDCl_3_, 150 MHz) of spectaline (**1**)

Figure S3: Enlarged ^13^C NMR spectrum (DMSO-*d_6_*+CDCl_3_, 150 MHz) of spectaline (**1**)

Figure S4: Full ^1^H NMR spectrum (DMSO-*d_6_*+CDCl_3_, 600 MHz) of spectaline (**1**)

H-14’

Figure S5: Enlarged ^1^H NMR spectrum (DMSO-*d_6_*+CDCl_3_, 600 MHz) of spectaline (**1**)

- Compound **2 (***Iso*-6-cassine**)**

White amorphous solid ^13^C NMR (150 MHz, DMSO-*d_6_*+CDCl_3_): *δ* (ppm) 209.0 (C-11’), 64.5 (C-3), 56.7 (C-6), 55.5 (C-2), 43.3 (C-10’), 33.2 (C-1’), 30.1 (C-4), 29.0 – 29.4 (C-2’-8’), 23.7 (C-9’), 22.5 (C-5), 16.0 (C-1’’). ^1^H NMR (600 MHz, DMSO-*d_6_*+CDCl_3_): *δ* (ppm) 8.02 (1H, s, H-1), 5.42 (1H, brs, OH-3), 3.73 (1H, s, H-3), 3.16 (1H, s, H-2), 2.95 (2H, s, H-6); 2.39 (H-10’), 2.06 (3H, s, H-12’), 1.80 (1H, dd, *J* = 18.0 and 6.0 Hz, H-4*α*), 1.65 (2H, m, H-5), 1.61 (1H, m, H-1’*α*), 1.57 (1H, dd, *J* = 18.0 and 6.0 Hz, H-4*β*), 1.45 (1H, m, H-1’*β*), 1.44 (2H, m, H-9’), 1.21-1.25 (14H, brs, H-2’-8’), 1.20 (3H, d, *J* = 12.0 Hz, H-1”).


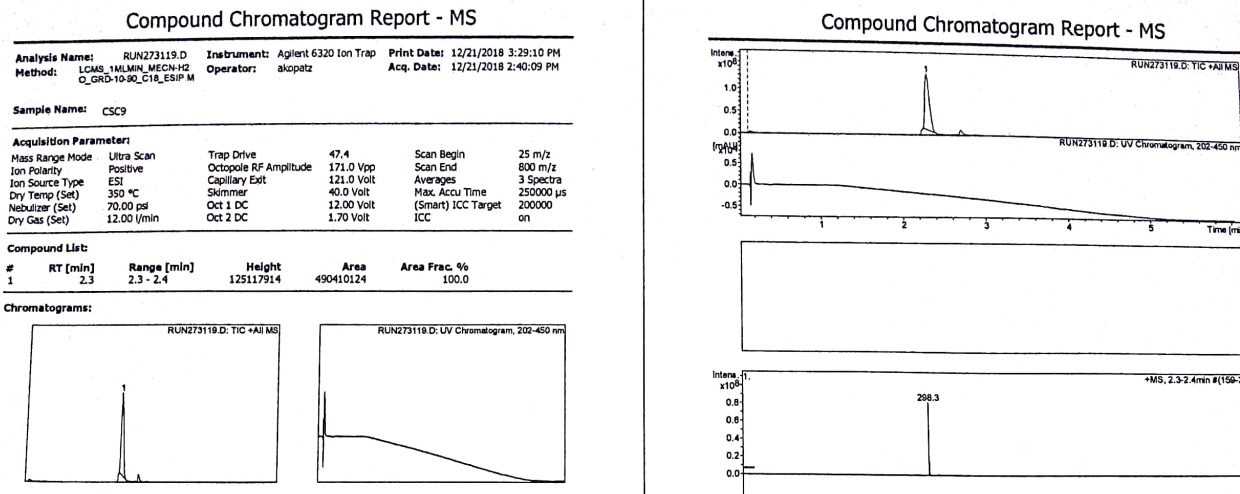


Figure S6: Full LC-MS profile of *Iso*-6-cassine (**2**)


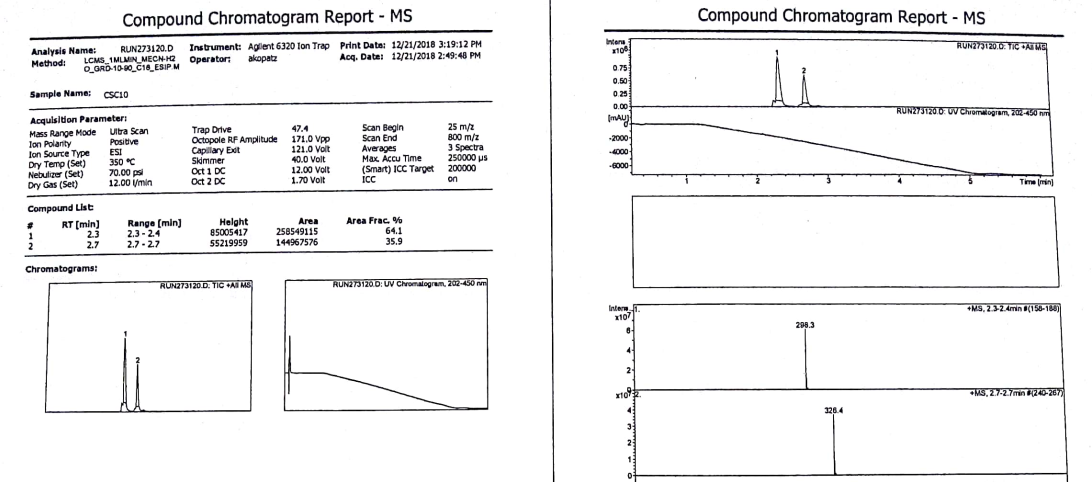


Figure S7’: Full LC-MS profile of CSC10, mixture of spectaline (**1**) and *Iso*-6-cassine (**2**)

Figure S8: Full ^1^H NMR spectrum (DMSO-*d_6_*+CDCl_3_, 600 MHz) of CSC10, mixture of spectaline (**1**) and *Iso*-6-cassine (**2**)

Figure S9: Full ^13^C NMR spectrum (DMSO-*d_6_*+CDCl_3_, 600 MHz) of CSC10, mixture of spectaline (**1**) and *Iso*-6-cassine (**2**)

- Compound **3 (**3-*O*-methyl-*chiro*-inositol**)**

White amorphous solid ^13^C NMR (150 MHz, DMSO-*d_6_*): *δ* (ppm) 84.3 (C-1), 73.0 (C-6), 72.8 (C-4), 72.5 (C-3), 71.3 (C-5), 70.5 (C-2), 60.1 (C-7),  ^1^H NMR (600 MHz, DMSO-*d_6_*): *δ* (ppm) 4.75 (OH-4), 4.66 (OH-3), 4.55 (OH-6), 4.50 (OH-2), 4.39 (OH-5), 3.63 (1H, m, H-4), 3.62 (1H, m, H-3), 3.50 (1H, m, H-2), 3.44 (3H, s, H-7), 3.43 (1H, dd, *J* = 6.0 and 3.0 Hz, H-5), 3.32 (1H, m, H-6), 3.00 (1H, t, *J* = 6.0 Hz, H-1).

Figure S10: Full ^13^C NMR spectrum (DMSO-*d_6_*, 150 MHz) of 3-*O*-methyl-*chiro*-inositol (**3**)

Figure S11: Full^1^H NMR spectrum (DMSO-*d_6_*, 600 MHz) of 3-*O*-methyl-*chiro*-inositol (**3**)

- Compound **4** (monobehenin)

Yellow amorphous solid ^13^C NMR (150 MHz, C_5_D_5_N): *δ* (ppm) 173.7 (C-1), 70.7 (C-2’), 66.7 (C-1’), 64.1 (C-3’), 34.2 (C-2), 29.8 - 31.8 (C-4/C-20), 25.2 (C-3), 22.8 (C-21), 14.3 (C-22). ^1^H NMR (600 MHz, C_5_D_5_N): *δ* (ppm) 4.66 (1H, dd, *J* = 18.0 and 6.0 Hz, H-1’*α*); 4.59 (1H, dd, *J* = 18.0 and 6.0 Hz, H-1’*β*), 4.38 (1H, m, H-2’), 4.06 (2H, d, *J* = 6.0 Hz, H-3’), 2.29 (2H, t, *J* = 6.0 Hz, H-2), 1.63 (2H, m, H-3), 1.18 - 1.20 (36H, brs, H-4-20), 1.15 (2H, m, H-21), 0.80 (3H, t, *J* = 6.0 Hz, H-22).


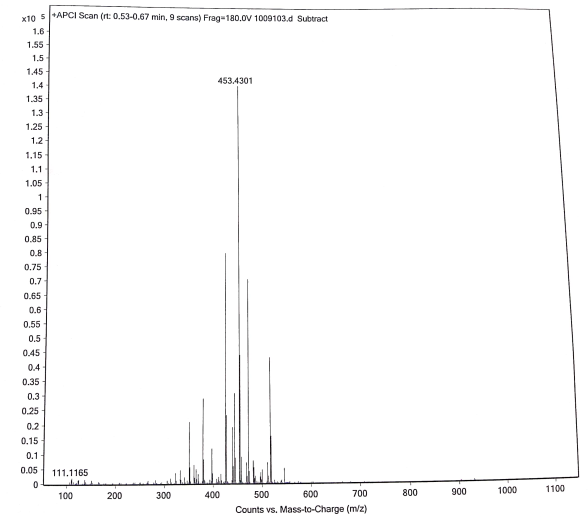


Figure S12 : Full APCI mass spectrum of compound **4**

Figure S13: Full ^13^C NMR spectrum (C_5_D_5_N, 150 MHz) of monobehenin (**4**)

Figure S14: Full^1^ H NMR spectrum (C_5_D_5_N, 600 MHz) of monobehenin (**4**)

- Compound **5 (**arachidic acid**)**

White amorphous solid ^1^H NMR (600 MHz, CDCl_3_): *δ* (ppm) 2.26 (2H, t, *J* = 7.0 Hz, H-2), 1.57 (2H, brq, *J* = 6.0 Hz, H-3), 1.20 – 1.26 (42H, brs, H-4/ C-26), 0.85 (3H, t, *J* = 6.0 Hz, H-27).


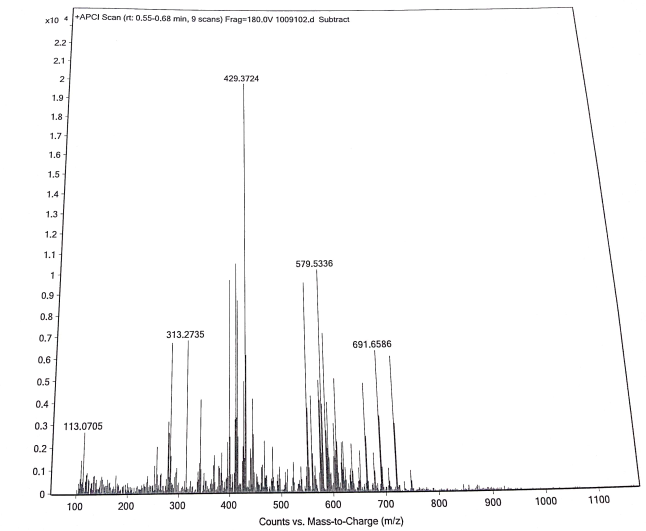


Figure S15 : Full APCI mass spectrum of compound **5**

Figure S16: Full^1^H NMR spectrum (CDCl_3_, 600 MHz) of arachidic acid (**5**)

- Compound **6 (***β*-sitosterol 3-*O*-*β*-D-glucopyranoside)

White powder.^13^C NMR (DMSO-*d_6_*): 11.7, 11.9, 18.8, 19.0, 19.2, 19.8, 21.1, 23.2, 24.3, 26.1, 28.3, 29.2, 30.0, 31.8, 31.9, 34.0, 36.2, 36.7, 37.2, 39.1, 39.7, 42.2, 45.8, 50.1, 56.0, 56.6, 62.6, 71.4, 75.1, 77.8, 78.4, 102.4, 121.7, 140.7. ^1^H NMR (DMSO-*d_6_*): 5.25 (1H, d, *J* = 4.6 Hz), 4.97 (1H, d, *J* = 7.7 Hz), 4.48 (1H, dd, *J* = 2.1, 11.7 Hz), 4.33 (1H, dd, *J* = 5.2 and 11.7 Hz), 4.23 (1H, m), 4.21 (1H, m), 3.97 (1H, pseudo-t, *J* = 8.2 Hz), 3.90 (1H, m), 3.85 (1H, m), 0.55 (3H, s), 0.83 (3H, s), 0.88 (3H, d, *J* = 6.4 Hz), 0.76 (3H, d, *J* = 6.9 Hz), 0.77 (3H, d, *J* = 7.8 Hz), 0.80 (3H, t, *J* = 7.6 Hz).

**
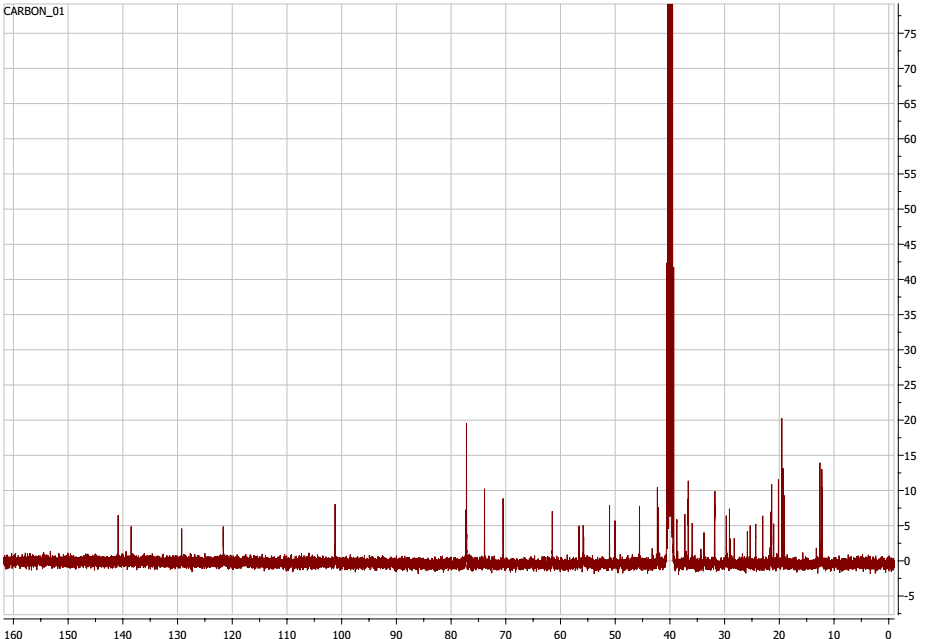
**

Figure S17: Full ^13^C NMR (DMSO-*d_6_*, 100 MHz) of compound **6**


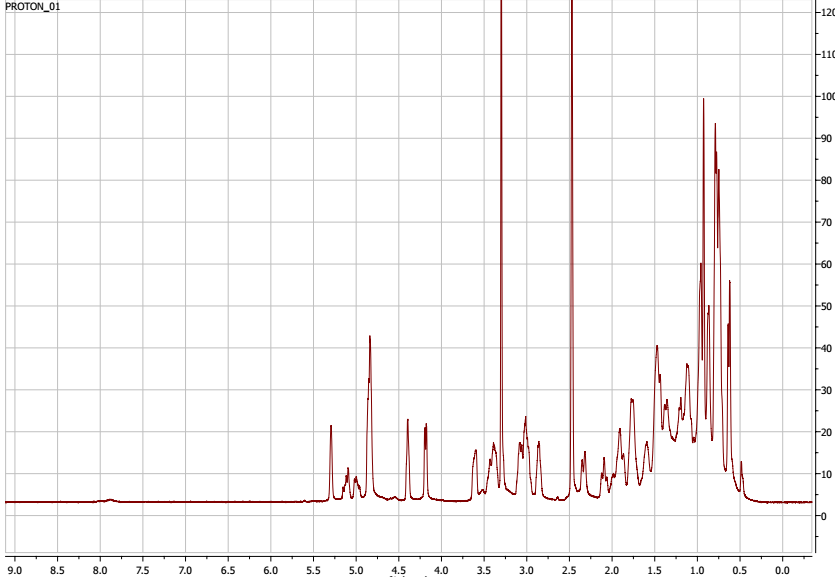


Figure S18: Full ^1^H NMR (DMSO-*d_6_*, 400 MHz) of compound **6**
